# Supramolecular solvent-based all-in-one extractions for comprehensive suspect screening of chemicals in food contact materials

**DOI:** 10.1007/s00604-025-07216-8

**Published:** 2025-06-03

**Authors:** Laura García-Cansino, Noelia Caballero-Casero, María Ángeles García, María Luisa Marina, Soledad Rubio

**Affiliations:** 1 Departamento de Química Analítica, Química Física E Ingeniería Química, Universidad de Alcalá, Ctra. Madrid-Barcelona Km. 33.600, Alcalá de Henares, Madrid 28871 Spain; 2 Department of Analytical Chemistry, Institute of Chemistry for Energy and the Environment, Universidad de Córdoba, Anexo Marie Curie, Campus de Rabanales, 14071 Córdoba, Spain; 3 Instituto de Investigación Química Andrés M. del Río, Universidad de Alcalá, Ctra. Madrid-Barcelona Km. 33.600, Alcalá de Henares, Madrid 28871 Spain

**Keywords:** Suspect screening, Food contact materials, Supramolecular solvents, Non-intentionally added substances, Intentionally added substances, All-in-one extraction, Liquid chromatography-high resolution mass spectrometry

## Abstract

Only a minimal fraction of the food contact chemicals (FCCs) estimated to be present in food contact materials (FCMs) have been ever detected (~ 3000 out of 100,000). Identifying as many FCCs as possible is imperative for evaluating their impact on human health. Supramolecular solvent (SUPRAS)-based all-in-one extractions are proposed for the first time for sample treatment of FCMs and combined with liquid chromatography high-resolution mass spectrometry for developing comprehensive suspect screening of FCCs. SUPRASs featuring inverted hexagonal and sponge-like nanostructures were investigated for this purpose. Those produced from 1-decanol in ethanol–water media, behaving as restricted access materials for macromolecules, showed excellent performance in terms of interference removal, reproducibility and number of features detected. Sample treatment was simple (only vortex-shaking and centrifugation is required), fast (FCC extraction and FCM cleanup were integrated in a single step) and sustainable (only 100 µL of SUPRAS per sample is required). An in-house suspect list containing 1389 chemicals was developed for FCCs identification. The approach developed was applied to determine  18 plastic- and Tetra-Brik-based FCMs. Twenty-nine FCCs (9 intentionally and 20 non-intentionally added substances; IAS/NIAS) belonging to eight chemical classes were identified. These FCCs featured a wide polarity range (log P − 5.2 to 9.8), thus proving the ability of SUPRASs to develop comprehensive extraction of FCCs and increase the chance of raising the number of FCCs identified (e.g., 18 out of the 29 FCCs found in this study have not been previously reported in FCMs).

**Supplementary Information:**

The online version contains supplementary material available at 10.1007/s00604-025-07216-8.

## Highlights

• SUPRASs are used for all-in-one extraction of food contact chemicals (FCCs).

• A SUPRAS-LC-HRMS approach is developed for the suspect screening of FCCs.

• 29 chemicals (log P −5.2 to 9.8) are identified in Plastic and Tetra Brik packages.

• 18 of the chemicals identified are firstly reported in food contact materials (FCMs).

• SUPRASs are valuable for addressing the wide chemical diversity present in FCMs.

**Graphical Abstract:**

**Supplementary Information:**

The online version contains supplementary material available at 10.1007/s00604-025-07216-8.

## Graphical abstract

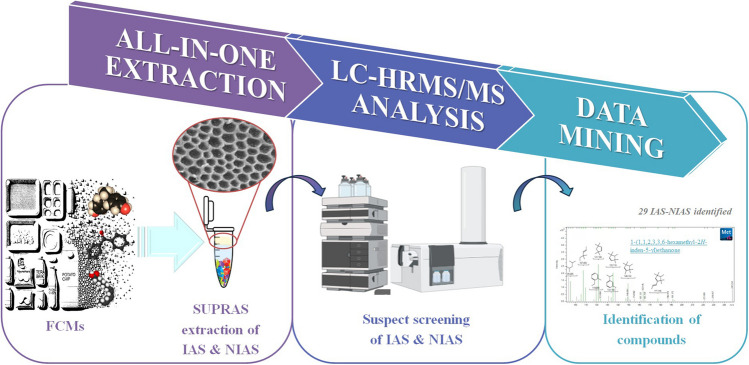

## Introduction

Food contact materials (FCMs), essential for enabling the current food system, contain hazardous components that can migrate into food and represent a global concern for human health [1]. A database containing 12.285 food contact chemicals (FCCs) whose presence is authorized in 18 FCMs has been compiled from many different regulations set forth in Europe, the USA, China, Japan, and the Mercosur region [2]. This database contains intentionally added “authorized substances” (IAS), that are permitted at specific migration limits in FCMs such as plastics, coatings, rubbers, paper/board, adhesives, and printing inks. The IAS are added to materials to impart different specific properties and include monomers, antioxidants, lubricants, surfactants, and light stabilizers [3]. Additionally, there is an infinity of other chemicals in FCMs, called “non-intentionally added substances” (NIAS), most of which remain unknown [4]. They include degradation products, impurities of raw materials, reaction byproducts, and contaminants from recycling processes.

In a recent study, Geueke et al. have compiled a database of 2881 migrating and extractable FCCs (FCCmigex) reported in 1210 studies that represents the most comprehensive overview of the empirical data available to date in this field [5]. Some interesting conclusions of this study were that around 65% of the 2881 detected FCCs were NIAS and that only a minimal fraction of the 12.285 IAS (~ 9.2%) has been ever detected. On the other hand, it has been estimated that FCMs could contain up to 100.000 FCCs [6]. So, it seems that the number of IAS/NIAS detected so far only represents the tip of the iceberg and that efforts should aim to have a more complete picture of chemicals in FCMs, in order to thoroughly assess their impact on human health [7].

Traditionally, the analysis of IAS and predicted NIAS in FCMs has predominantly relied on targeted methods [8]. Recently, suspect and non-targeted analysis based on chromatographic techniques coupled with high-resolution mass spectrometry, particularly liquid chromatography (LC-HRMS), have become the primary choices for the identification of IAS/NIAS with a wide range of physical–chemical properties [9]. Valuable assets of these technologies include very precise mass measurements, good sensitivity in data acquisition, and wide dynamic range, providing a high number of chemical information for the detection of a large number of compounds without previous information [10]. However, most suspect and non-targeted analyses of FCCs are not sufficiently comprehensive because many chemicals are lost during sample preparation or are not identified after data processing [11, 12]. So, a key aspect that remains unsolved in this field is the development of unbiased sample preparation methods able to efficiently transfer a wide-polarity range of FCCs from the FCMs to the separation/detection system in a single step [13].

More than half of the FCC data reported so far have been obtained by the direct extraction of FCMs with a variety of organic solvents [5]. Non-polar compounds are mainly extracted with hexane or ethyl acetate, while the polar ones are extracted with methanol or ethanol [11]. In general, repetitive extractions (e.g., 15 × 3 mL of methanol per gram of sample [14] or 10 × 3 mL of hexane per 0.2 g of sample [12]), using single or combined auxiliary energies (i.e., ultrasonication, pressure, high temperature), followed by sample clean-up (Quechers, SPE) or evaporation and reconstitution with an LC-compatible solvent, are the most common sample processing strategies [8]. Unfortunately, in addition to requiring costly, labor-intensive and non-environmentally-friendly procedures, the polarity range of the solubilized compounds in solvent-based extractions is highly dependent on the nature of the solvent, thus highlighting the need to develop more innovative approaches for setting up more comprehensive FCM analyses. To confront this challenge, analytical methods able to identify as many as possible IAS/NIAS through quick and single procedures would be desirable.

In this paper, we aimed to apply the concept of *all-in-one extraction*, based on supramolecular solvents (SUPRASs), to develop a comprehensive sample processing suitable for the suspect screening of chemicals in FCMs using LC-HRMS. SUPRASs are nanostructured liquids produced from colloidal solutions of amphiphiles through a bottom-up strategy driven by the balance of non-covalent repulsive and attractive interactions [15]. Their potential for achieving all-in-one extractions in chemical exposomics has been recently discussed [16]. All-in-one extractions have been defined as extraction methodologies able to extract multiclass substances covering a wide range of polarities and physicochemical properties in an efficient, cost-effective and sustainable way, and provide solvent extracts ready for direct analysis with minimal manipulation [16].

There are three valuable assets of SUPRASs for developing all-in-one extractions able to set up comprehensive suspect analyses of chemicals in FCMs [15]. First, SUPRAS is suitable for the efficient solubilization of chemicals in a wide polarity range, unlike conventional solvents, thanks to the presence of two regions with different polarities in their nanostructures. These regions provide mixed-mode mechanisms for solute solubilization that together with the high concentration of amphiphiles in the SUPRAS (0.1–1 mg µL^−1^) further enhances the availability of binding sites for analytes, allowing for the use of small volumes of SUPRAS while maintaining high extraction efficiency. Secondly, SUPRAS ability to develop fast microextractions under mild conditions, which results from the combination of their discontinuous character (they are formed by micrometric coacervate droplets that speed up mass transfer), and the huge number and variety of binding sites. Thirdly, the possibility of tailoring both SUPRAS properties and nanostructures has enabled the development of solvents that behave as restricted access materials (SUPRAS-RAM) that can remove matrix macromolecules (proteins, carbohydrates) during extraction, thus facilitating the production of clean extracts [13].

In this research, four SUPRASs featuring different chemical compositions and nanostructures were investigated for the suspect screening of IAS and NIAS (1389 chemicals, log P from − 5.2 to 26) by LC-HRMS with the aim of developing a comprehensive sample processing for their extraction and identification from eight types of plastic- and Tetra Brik-based FCMs. The SUPRAS-based sample treatment was optimized and the method was evaluated in terms of matrix effects, and reproducibility. The data analysis was based on the prioritization and reduction of the number of features (e.g., intensity threshold, signal-to-noise threshold, and mass range restrictions) prior to the annotation of FCCs using an in-house database [17].

## Experimental

### Chemicals

Both the organic solvents tetrahydrofuran (THF), ethanol (ETOH), methanol (MeOH), isopropanol (ISOPrOH), and sodium hydroxide (NaOH) were purchased from Panreac (Barcelona, Spain). Ultra-high-quality water was obtained from a Milli-Q water purification system (Millipore, Madrid, Spain). 1,2-decanediol, 1-decanol, 1-octanol, sodium chloride (NaCl), and formic acid (Fac) were supplied by Sigma-Aldrich (Barcelona, Spain). For the experiments, a mixture of four labelled-Internal Standards (triphenyl phosphate-d_15_ (TPHP-d_15_), (diphenyl phosphate-d_10_ (DPP-d_10_), bisphenol S-d_8_ (BPS-d_8_), and 2,4,6-tribromophenol-^13^C_6_ (TrBrPh-^13^C_6_)) was prepared at 500 µg L^−1^ in MeOH. Table S1, in Supplementary Information (SI), displays chemical information on these internal standards (IS).

### Samples

Eighteen foodstuff samples contained in FCMs based on plastic- (*n* = 16) and Tetra Brik (*n* = 2) were acquired from local supermarkets in Córdoba (Andalusia, Spain) between November and December 2023. Plastic-based FCMs belonged to the following categories: polyethylene terephthalate (PET), including also recycled PET (rPET); high-density polyethylene (HDPE); low-density polyethylene (LDPE); polypropylene (PP); polystyrene (PS); and polycarbonate (PC). Tetra Brik containers, made up of multilayers (cardboard, aluminum and plastic), were labelled as mixed FSC (FOREST Stewardship Council), which refers to products arising from FSC-certified forest, recycled materials and/or FSC-controlled wood [18]. The containers (*n* = 18) were rinsed twice with distilled water in order to remove potential compounds and remains from foodstuffs, and subsequently cut into pieces of approximately 1 cm^2^ for extraction.

### Synthesis of SUPRASs

Four types of SUPRASs were investigated for extraction of FCCs from the selected FCMs. They were produced from colloidal solutions of 1,2-decanediol, 1-decanol or 1-octanol through spontaneous coacervation processes. Figure S1 shows a general schematic of SUPRAS synthesis and Table S2 gives the relative proportions of reagents and solvents used in SUPRAS production. The amphiphile was solubilized in the solvent to produce the colloidal solution and then, the coacervation-inducing agent was added and the mixture was manually stirred for approximately 30 s and centrifuged at 2400* g* for 15 min. This resulted in two immiscible liquid phases, with the SUPRAS and the equilibrium solution forming the top and bottom layers, respectively. Then, each phase was separately transferred into hermetically sealed glass vials, and they were stored at 4 °C until use.

### Optimization of SUPRAS-based extraction

**Fig. 1:**
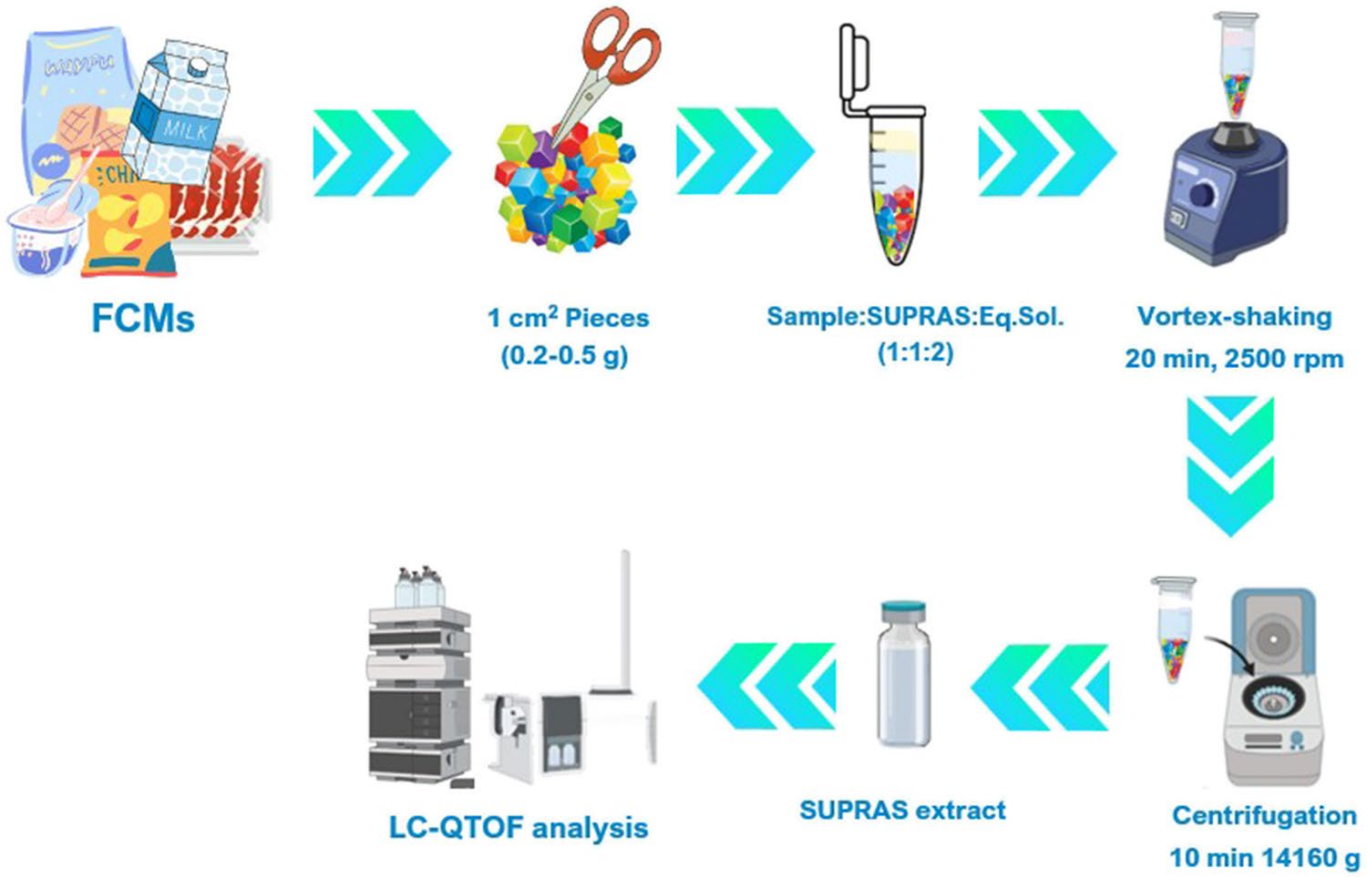
Scheme of the general procedure for the suspect screening of FCCs in FCMs using the SUPRAS-LC-ESI-HRMS approach

Tetra Brick, which is a multilayer material, was selected as a model FCM to optimize the extraction procedure. Selection among the four SUPRASs investigated (Table S2) was based on the number of labelled IS detected and identified (i.e., detection frequency, % DF), the matrix effects (% ME) and reproducibility (expressed as relative standard deviation of the calculated % ME, % RSD) obtained for the labelled IS, as well as the number of features extracted. Then, for the SUPRAS selected, chemical composition and sample/SUPRAS ratio were optimized. Experiments were carried out into Safe-Lock 2 mL microtubes from Eppendorf Ibérica (Madrid, Spain). Four spherical glass beads (3 mm diameter) were introduced in the microtube to facilitate sample dispersion during extraction. To the sample (1 cm^2^ pieces of FCM corresponding to 0.2–0.5 g) it was added the SUPRAS (0.2–0.5 mL) and the equilibrium solution (0.4–1 mL). Then, the mixture was vortex-shaken (2500 rpm for 20 min) and centrifuged (10 min at 14,160* g*). In the case of polystyrene-based containers, the volume of equilibrium solution was incremented until the total wetting of the 1 cm^2^ pieces. Finally, the SUPRAS extract was transferred to an LC vial and enriched with 50 ng mL^−1^ of the IS mixture prior to the analysis by LC-QTOF. Figure 1 depicts a general scheme of the optimized and recommended SUPRAS-based sample treatment for the suspect screening of IAS/NIAS in FCMs.

### LC-QTOF-MS/MS analysis

The labelled-ISs and IAS/NIAS were separated and detected using an Elute UHPLC Pump HPG 1300 coupled to a TIMS TOF mass spectrometer (Bruker Daltonics, Bremen, Germany) equipped with an electrospray ionization source (ESI). The chromatographic separation was carried out on a Restek Roc C_18_ column (100 × 3.0 mm, particle size 3 µm), along with a C_18_ Security Guard Cartridge (Phenomenex C18 4.0 × 2 mm) from Phenomenex at 40 °C. Formic acid was used as mobile phase modifier for positive ionization mode to efficiently promote protonation and enhance sensitivity, whereas ammonium fluoride was expected to improve deprotonation efficiency and ion stability in negative mode. Thus, the mobile phase consisted of H_2_O (A) and MeOH (B), both containing 0.1% formic acid for the positive ionization mode (ESI +). For the negative ionization mode (ESI −), the mobile phase consisted of 0.5 mM NH_4_F in aqueous solution (A) and MeOH (0.5 mM NH_4_F) (B). The flow rate was 0.25 mL/min and the volume injection was 10 µL for both ESI + and ESI −. The chromatographic conditions were optimized based on the number of detected and identified labelled-ISs and considering key chromatographic parameters, including peak width, resolution, and capacity factor (k), calculated for these ISs. Optimal separation was achieved with the following linear gradients: in positive mode, 65% A in initial conditions, 0% A (0–0.1 min), 45% A (0.1–8 min), 65% A (8–9 min), 20% A (9–15 min), 65% A (15–25 min), 55% A (25–27 min), 40% A (27–28 min), and 0% A (28–30 min); and in negative mode, 65% A in initial conditions, 55% A (0.1–8 min), 45% A (8–9 min), 40% A (9–15 min), 20% A (15–21 min), 0% A (21–27 min), and 65% A (27–30 min).

Mass spectrometric analyses were carried out under optimal ion source parameters: end-plate offset, 500 V; capillary voltage, ± 4500 V; nebuliser gas pressure, 58 psi; dry gas, 10 L/min; dry temperature, 220 °C. For suspect screening analyses data-dependent acquisition (DDA) mode, specifically the auto-MS/MS acquisition mode, was applied. Auto-MS/MS mode is based on the automatic selection of the three precursor ions most intense per cycle for fragmentation. For the recording of the MS and MS/MS spectra, a spectrum rate of 4 Hz was applied in the range of 50–1900 m/z units. A collision energy ramp was used (from 0 to 20 eV) and spectra were averaged. The active exclusion mode was enabled to prevent the repetitive acquisition of MS/MS spectra for the same precursor ion and was set at 3 cycles. Data acquisition was carried out with *otofControl 6.0 software* (Bruker), and stored in line mode before exporting for further analysis.

All samples of the plastic-based FCMs were analyzed under the described chromatographic and acquisition conditions. The analytical method was also evaluated in terms of precision (evaluated for the ISs employed as control), both intrabatch and interbatch, measured in RSD (%), and in terms of matrix effect expressed as signal suppression or enhancement (SSE, %).

### QA/QC measurements

Some of the compounds classified as IAS, and of course the potential compounds considered as NIAS, may be present in other materials or even exhibit a ubiquitous nature. Therefore, it is crucial to reduce or eliminate potential sources of contamination during sample analysis. For this purpose, various QA/QC measures were implemented.

Work surfaces were cleaned daily with methanol, and paper was substituted with aluminum foil on the workbench. Additionally, priority was given to using lab glassware, which was previously rinsed with ethanol. When this was not feasible, single-use plastic laboratory materials were utilized, having been rinsed with ethanol beforehand. Furthermore, two procedural blanks containing labelled-ISs at the same concentration as the samples were included in each batch.

To achieve high performance of the LC-QTOF analytical method, several measures were also taken. Firstly, daily fresh mobile phases were used. The MS instrument was calibrated daily within the Working Mass Range using the calibration solution recommended by the supplier. A vial containing ISs solution mixture at 50 ng mL^−1^ was injected at the beginning and end of each batch of analyses and when a reduction in the signal was observed, the ion source was thoroughly cleaned and the batch was re-analyzed. In addition, one vial of MeOH was analyzed every eight injections in order to detect any carry-over effects or any contamination from the LC-QTOF-MS spare parts. All labelled IS-enriched samples were analyzed in duplicate to evaluate the performance of the analytical method.

### Data-analysis workflow

For data analysis, an in-house suspect list was developed. In the case of IASs, the analysis encompassed organic compounds explicitly regulated under European Union legislation [19]. Regarding NIASs, the focus was on classes of compounds classified as emerging concerns by NORMAN due to the limited understanding of their fate, behavior, and potential health effects. The suspect list encompassed 1389 compounds including IASs and NIASs, which belong to the following classes: additives (*n* = 367), emerging halogenated flame retardants (*n* = 796), emollients and emulsifiers (*n* = 14), personal care products (*n* = 51), plasticizers (*n* = 83), UV filters, stabilizers and inks (*n* = 48), and others (*n* = 30). For each compound included in the suspect list, relevant information was gathered from dedicated scientific literature and databases such as PubChem and NORMAN. Specifically, the data collected included chemical information such as the IUPAC name, MS-ready formula, monoisotopic mass, chemical classifications, CAS number, InChIKey, InChI, and canonical SMILES. Finally, to create a functional suspect list, the compiled Excel file was converted into a comma-separated values (CSV) format.

**Fig. 2:**
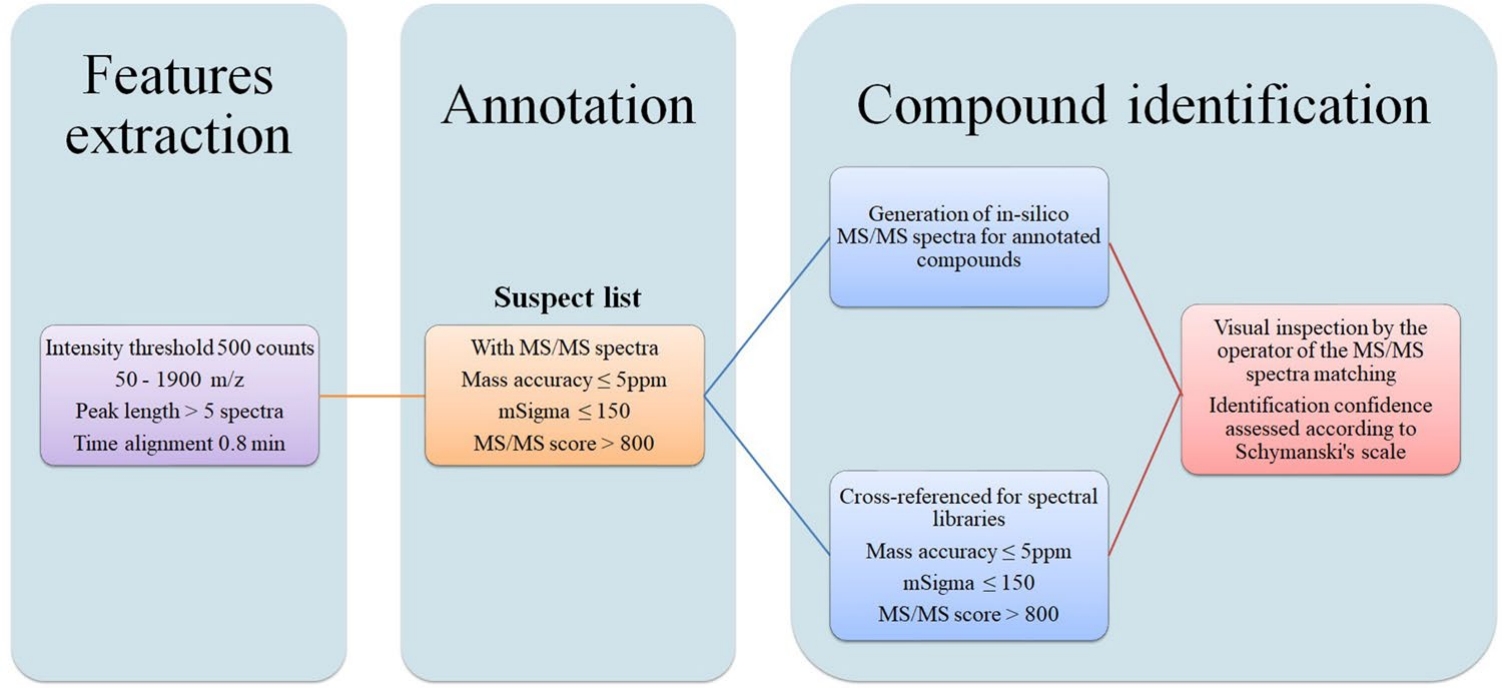
Schematic of the data-analysis workflow for compound identification

The workflow developed for data analysis utilizing a suspect screening approach is shown in Fig. 2. Firstly, feature extraction was carried out using the following parameters in the software *MetaboScape* 2023 (Bruker Daltonics): intensity threshold 500 counts, minimum peak length 10 spectra, minimum peak length (recursive) 5 spectra, retention time range 0.5 to 30 min, mass range 50 to 1900, perform MS/MS import and group by collision energy, ion deconvolution with EIC correlation of 0.8, MS recalibration auto-detect/auto-detect. Sample features were compared with those from procedural blanks, retaining only features detected in at least two samples and with an intensity at least three times higher than that of the procedural blanks. Annotation was conducted using the *in-house* suspect list. This process applied criteria including a mass tolerance of ± 5 ppm for parent ions and a matching score exceeding 800. Features from the samples were compared to those in procedural blanks, retaining only those present in at least two samples and exhibiting a fold change greater than 3 for further identification. Tentatively identified compounds with MS/MS spectra were flagged for additional analysis. Their MS/MS spectra were cross-referenced for public spectral libraries (i.e., MassBank Europe, MassBank of North America and PubChem). If no matches were found, MS/MS spectra were generated *in-silico* using dedicated software (MetFrag, v.2.1) and compared for compound spectra matching. Criteria of mass tolerance for product ions of ± 5 ppm and isotope abundance matching (expressed as mSigma) within ± 150 for both ionization modes between the measured and the predicted spectra were set. Only features with a matching score above 800 were selected and an operator manually reviewed all fragmentation spectra to validate the identifications. The confidence level of all features identified was determined following Schymanski’s scale. This evaluation incorporated additional parameters, such as retention time and compound polarity (logP), rather than relying solely on spectral data.

## Results and discussion

### SUPRAS selection

**Fig. 3:**
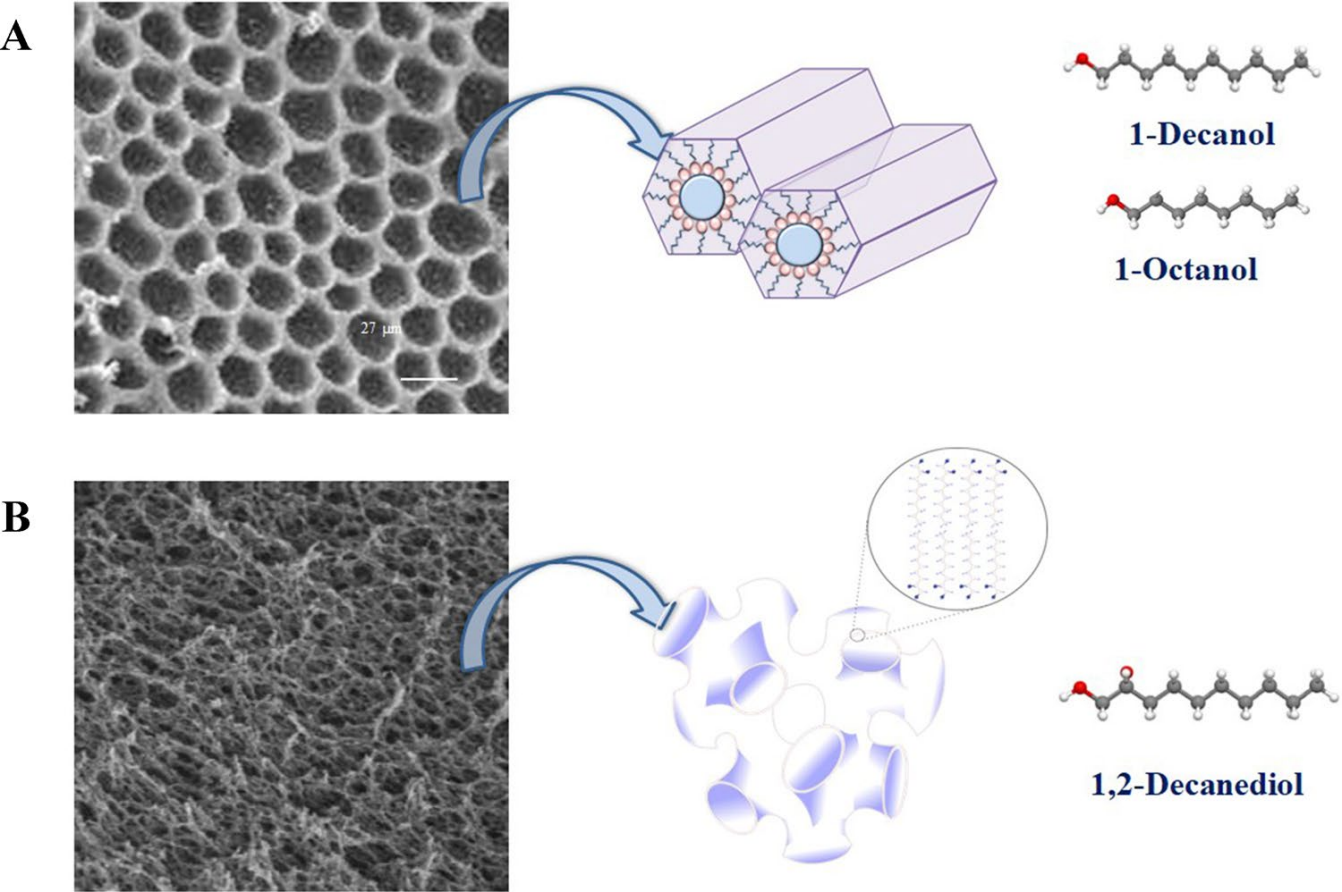
Electron micrographs and illustration of the nanostructures, as well as the amphiphiles investigated, for SUPRASs based on **A** alkanols and **B** alkanediols

The SUPRASs investigated for extraction of IAS and NIAS were prepared from colloidal solutions of alkanols [20] and alkanediols [21] using water or saline water as inducing-coacervation agents. (Table S2). Figure 3 displays the specific amphiphiles used in this study, as well as the electron microphotographs and illustrations of the nanostructures in which they arrange into the SUPRAS, namely, inverted hexagonal aggregates (Fig. 3A) and sponge-like morphologies (Fig. 3B). There were several reasons for testing these SUPRASs for the intended purpose (i.e., the screening of 1389 FCCs, log P − 5.2 to 26). First, alcohol groups do not ionize under typical ESI conditions, which makes these amphiphiles *transparent* in MS detectors. Second, although both alkanols and alkanediols produce SUPRASs with polar and non-polar microenvironments, they not only vary in their nanostructures (Fig. 3) but also in the extension of their polar region. Thus, the water content in alkanediol-based SUPRASs is quite constant and around 30–34%, independent of the composition of the synthesis solution [21]. In contrast, the water content in alkanol-based SUPRASs is highly dependent on the solvent percentage used during synthesis. While it increases with higher solvent percentages, it typically falls within the range of 4–15%. So, there is the possibility that both types of SUPRASs have different solubilization capacities for polar compounds. The third reason is that alkanol-based SUPRASs behave as restricted access materials (SUPRAS-RAM), which allow for the removal of proteins and carbohydrates in the extraction process, thus decreasing matrix interferences and having the possibility of integrating FCC extraction and FCM cleanup in a single step [13]. The RAM properties of alkanol-based SUPRASs derive from the size of the aqueous cavities of the inverted hexagonal aggregates (Fig. 3A), which are not big enough to accommodate polar macromolecules [20].

**Fig. 4:**
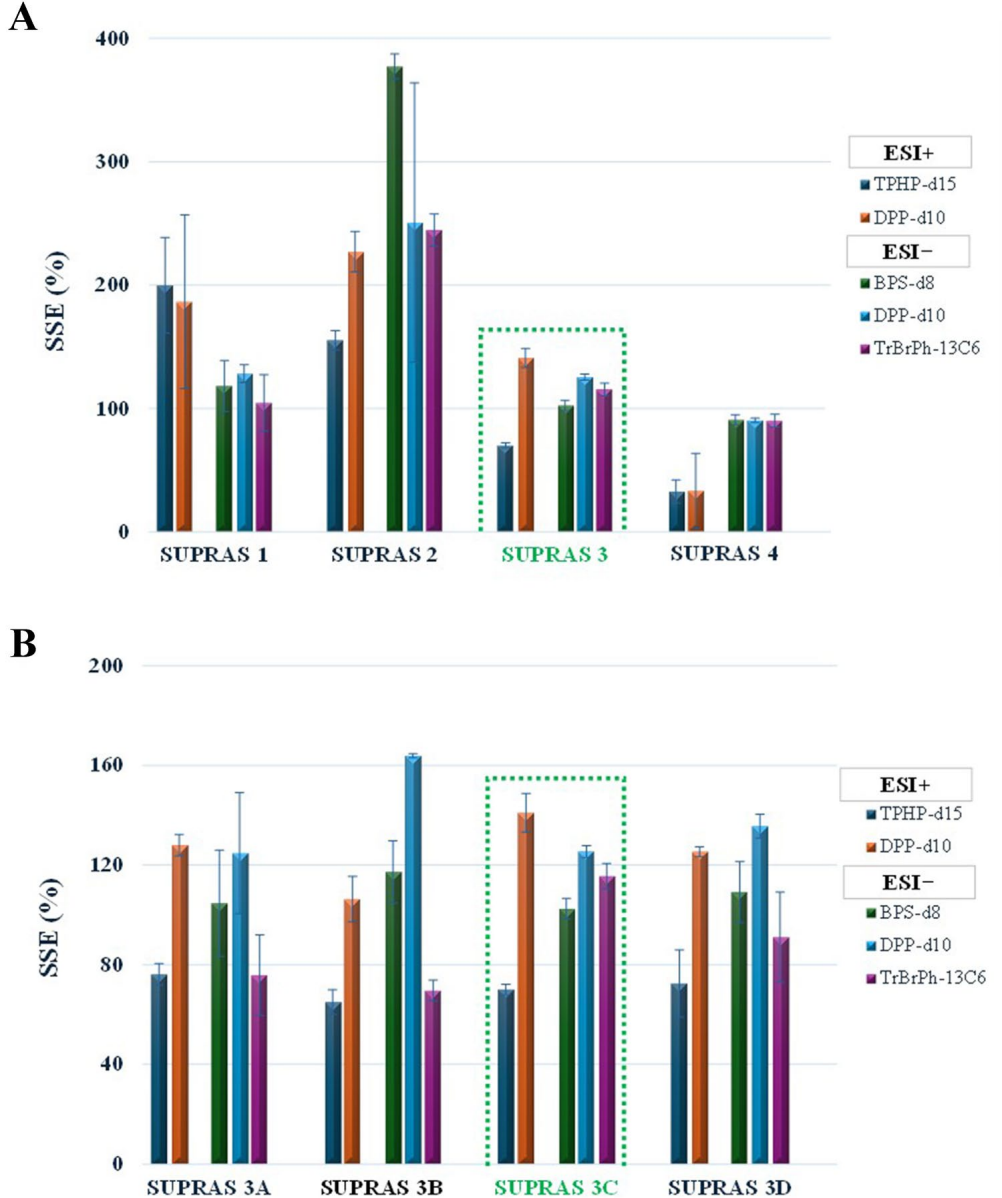
**A** Values of SSE (%) along with the standard deviation obtained for the labeled-IS in four types of SUPRAS synthesized under the following conditions. *SUPRAS 1:* 15% (w/w) 1,2-decanediol, 70% (w/w) NaCl 1 M, 15% (w/w) THF. *SUPRAS 2:* 15% (w/w) 1,2-decanediol, 70% (w/w) H_2_O, 15% (w/w) THF. *SUPRAS 3:* 10% (v/v) 1-decanol, 60% (v/v) H_2_O, 30% (v/v) EtOH. *SUPRAS 4:* 10% (v/v) 1-Octanol, 60% (v/v) H_2_O, 30% (v/v) THF. (B) Evaluation of SSE (%) for SUPRASs made up from 10% (v/v) 1-decanol synthesized under different conditions of 1-decanol/ethanol/water v/(v/v): *SUPRAS 3 A:* 10/10/80%; *SUPRAS 3B:* 10/20/70; *SUPRAS 3:* 10/30/60%; *SUPRAS 3 C:* 10/40/50%

Figure 4A shows the results obtained from the extraction of Tetra Brik samples with the four SUPRASs prepared as specified in the “Experimental” section and Table S2. Results for the labelled-IS selected, which were measured in both positive and/or negative ionization mode, were expressed as %SSE and their corresponding %RSD. These results clearly displayed that sponge-like SUPRASs (i.e., 1 and 2) were not globally efficient in the removal of matrix interferences for the measurement of the labelled-IS investigated. Only the SUPRAS based on 1,2-octanediol using saline water as the coacervation-inducing agent (SUPRAS 1, Table S2) was free of interferences for the ISs measured in the ESI negative mode. On the other hand, ionization suppression was observed in the ESI positive mode for SUPRAS-RAM extracts obtained from 1-octanol in a THF-water medium (i.e., SUPRAS 4). Good overall selectivity was obtained for the SUPRAS-RAM made up of 1-decanol in an ethanol–water medium (SSE values for the ISs were in the interval 70–140% with RSDs from 2.1 to 7.7%). Additionally, the number of features extracted from the Tetra Brik material was the highest compared with the rest of SUPRASs investigated. So, the SUPRAS 3 was consequently selected for the screening of FCCs in FCMs.

### Optimization of SUPRAS composition

The chemical composition of SUPRASs can be tailored by modifying the relative proportion of ingredients in the synthesis solution. In the case of decanol-based SUPRASs, the content of both ethanol and water in the SUPRAS increases as the percentage of ethanol in the colloidal solution does [16]. So, the influence of the chemical composition of the SUPRASs on their capacity for the extraction of FCCs from Tetra Brik samples was investigated by synthesizing SUPRASs with a constant concentration of 1-decanol (10%, v/v) while varying the ethanol content from 10 to 40% (v/v). The remaining volume was completed with water to reach 100% of the synthesis solution.

Figure 4B presents the SSE values, along with their corresponding RSDs, obtained for the labelled-ISs as a function of the chemical composition of the SUPRASs. On the whole, there were no significant differences in the SSE values obtained for the SUPRASs investigated, indicating that all of them behaved as restricted access materials and could integrate FCC extraction and FCM cleanup. Neither is there was overall significant difference in the number of features extracted. Better RSD values were globally obtained for SUPRAS 3 C, synthesized from decanol:ethanol:water at the relative proportions 10:30:60, expressed as % v/v, and this was the SUPRASs selected for further studies.

Regarding sample/SUPRAS ratio, it was selected the minimum volume of SUPRAS required to obtain a sufficient extract volume for at least two injections per sample in the LC-ESI-QTOF system (i.e., 100 µL). The volume of the equilibrium solution was shown to have no significant impact on the extraction process, as no substantial differences in the number of features were observed when compared to procedural blanks analyzed under the same conditions. Consequently, only the volume necessary to facilitate the collection of the SUPRAS extract was added.

### SUPRAS-LC-ESI-QTOF suitability

The 18 FCMs samples were spiked with labelled-IS prior to analysis by LC-ESI-QTOF. Before proceeding with data analysis, the presence of all labelled-ISs was verified by extracting the typical chromatogram for the m/z of each precursor ion using *Data Analysis*, v.5.3 software (Bruker Daltonics). The signal intensity for each IS had to exceed 10^3^ counts. Once this verification was complete, data analysis for IS was performed following the proposed Workflow (Fig. 2). All labelled ISs were positively identified in the analyzed samples with a mass accuracy of ± 5 ppm. Precision was calculated for each labelled IS in both ESI^+^ and ESI^−^ modes using their corresponding peak areas obtained from intrabatch and interbatch analyses. In ESI^+^, the intrabatch RSD (%) values were 18.3% for THPH-d_15_ and 2.7% for DPP-d^10^, while the interbatch RSD (%) values were 18.0% and 25.8%, respectively. In ESI^−^ mode, the intrabatch RSD (%) values were 13.5%, 4.5%, and 10.3% for BPS-d_8_, DPP-d_10_, and TrBrPh-^13^C_6_, respectively, whereas the interbatch RSD (%) values were 7.8%, 22.6%, and 9.7%, respectively.

Background contamination was monitored by analyzing procedural blanks and solvent injections. None of the identified and reported compounds (confidence levels 1–3) were detected in the procedural blanks or solvent injections. Additionally, no carry-over effects were observed in the solvent injections. Molecular features identified in each FCM-type sample were cross-checked between duplicate samples. Only features consistently present in both replicates were considered for identification. The QC results demonstrated stable and reproducible performance of the instrument, ensuring reliable results throughout the analysis.

### FCCs identified in plastic- and Tetra Brik-based FCMs

**Fig. 5:**
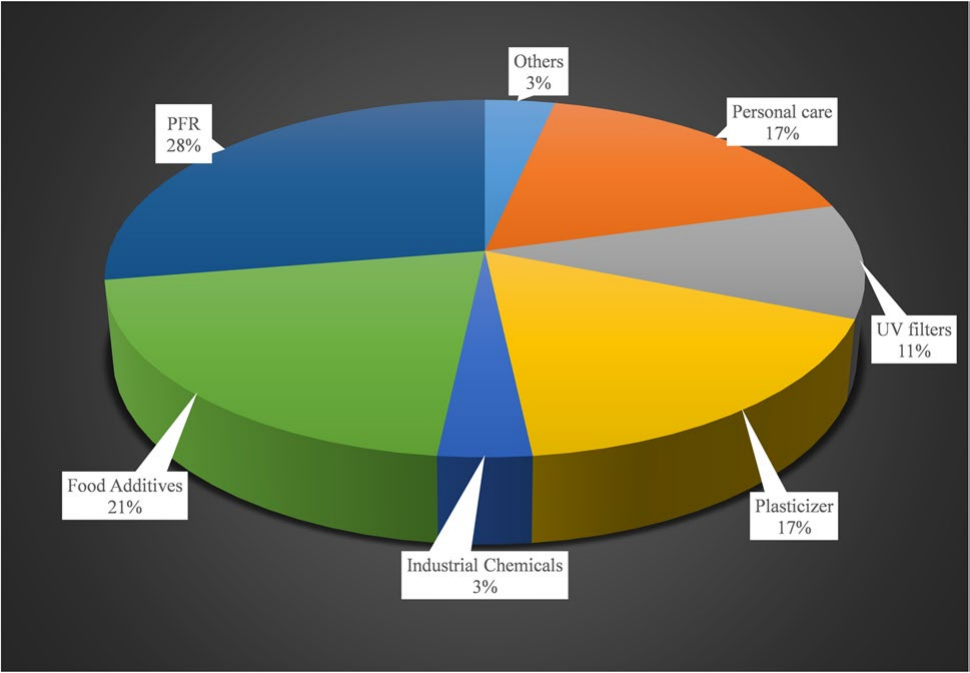
Pie chart showing the distribution of chemical groups identified in the FCM samples

A total of 29 compounds were tentatively identified across the 18 analyzed FCM samples, with their identification confirmed through visual inspection of spectra, achieving a confidence level of 2a-3 [22]. The 29 identified compounds were categorized into 8 different chemical classes, comprising 9 IAS, included in the EU regulation [19], and 20 NIAS. Table 1 summarizes the identified FCCs, along with details on their detection and identification, including experimental retention times, mass error, level of confidence, and detection frequency. Figure 5 illustrates the distribution of the identified compounds among the various chemical classes.

**Table 1:**
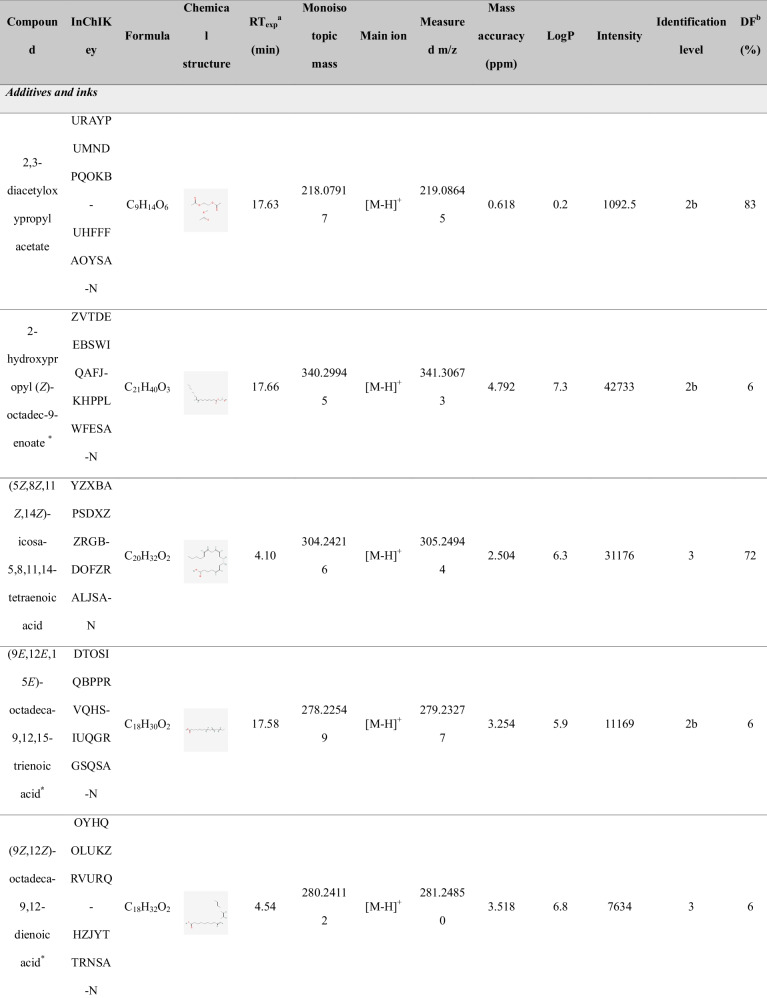

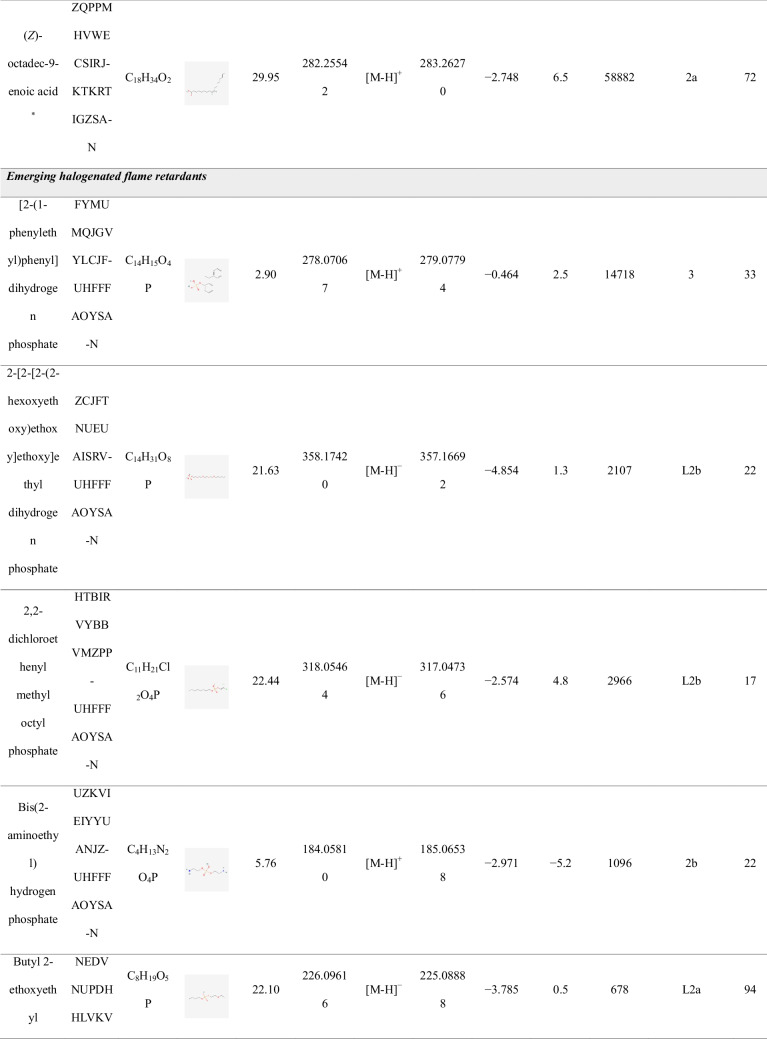

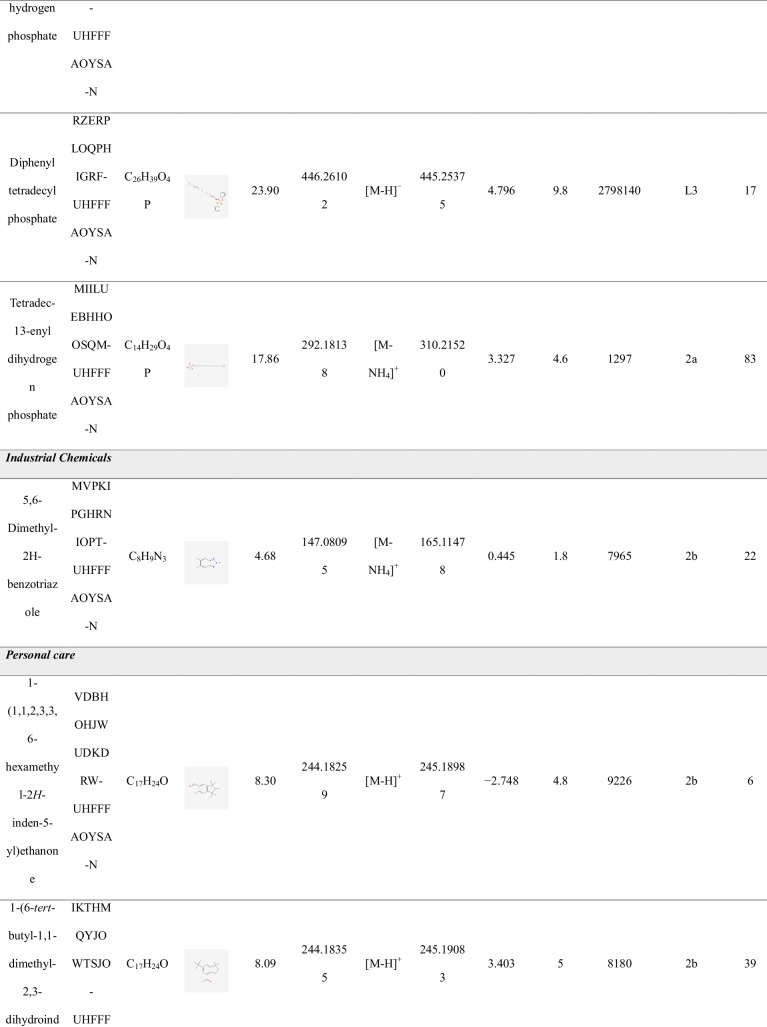

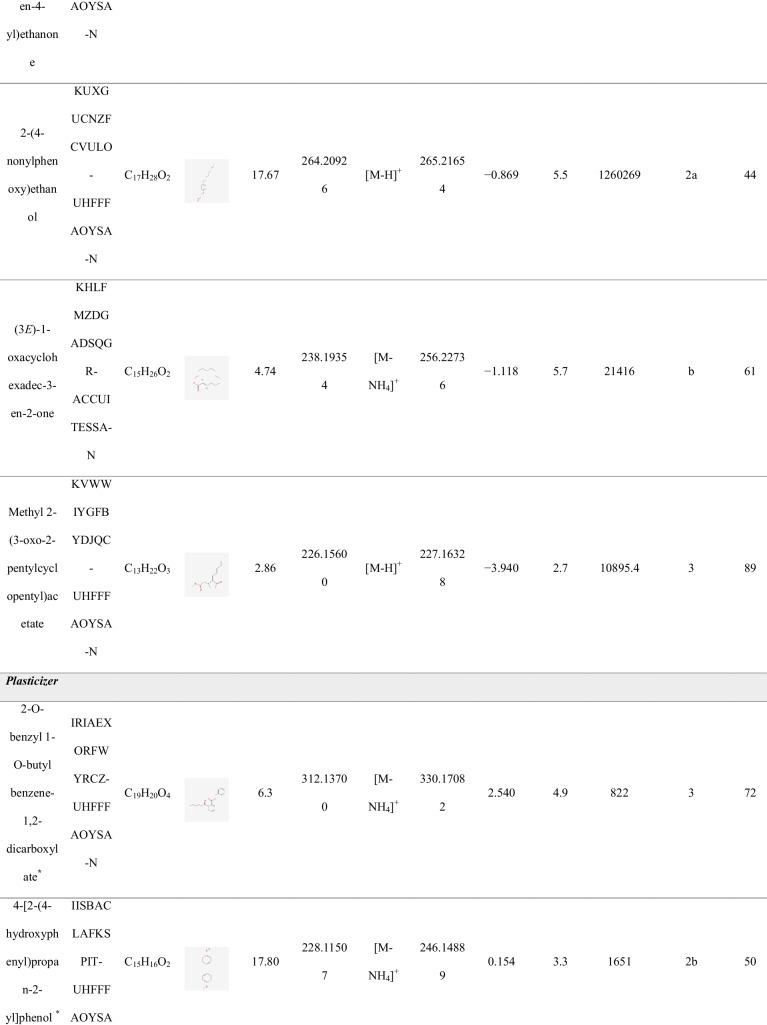

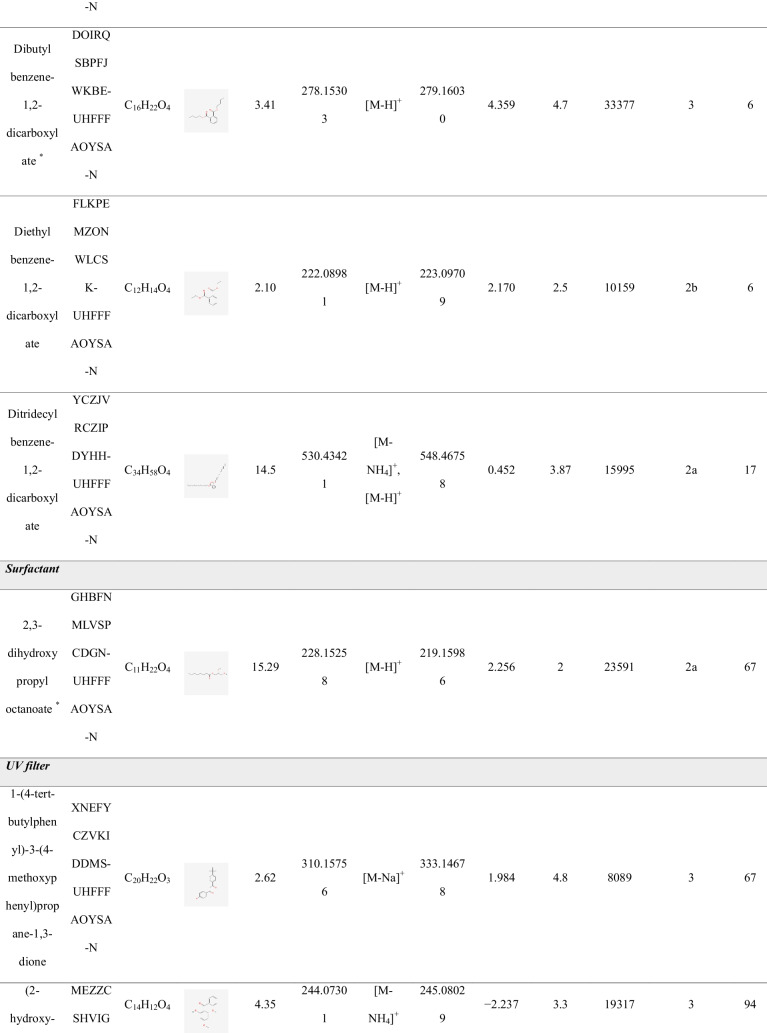

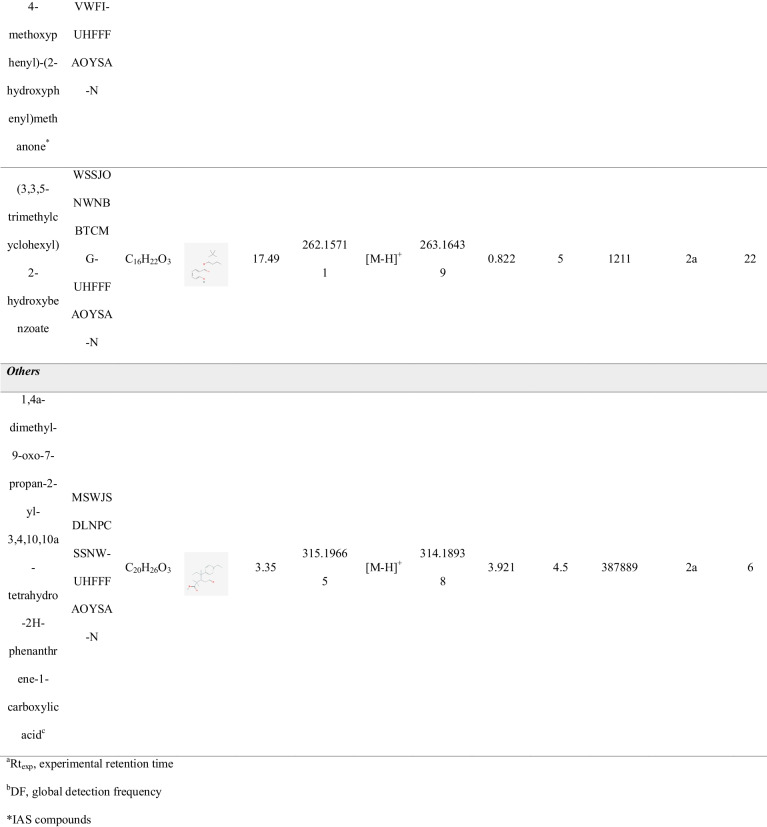
Compounds identified in the FCMs analyzed

^a^Rt_exp_, experimental retention time. ^b^DF, global detection frequency. *IAS compounds

**Fig. 6:**
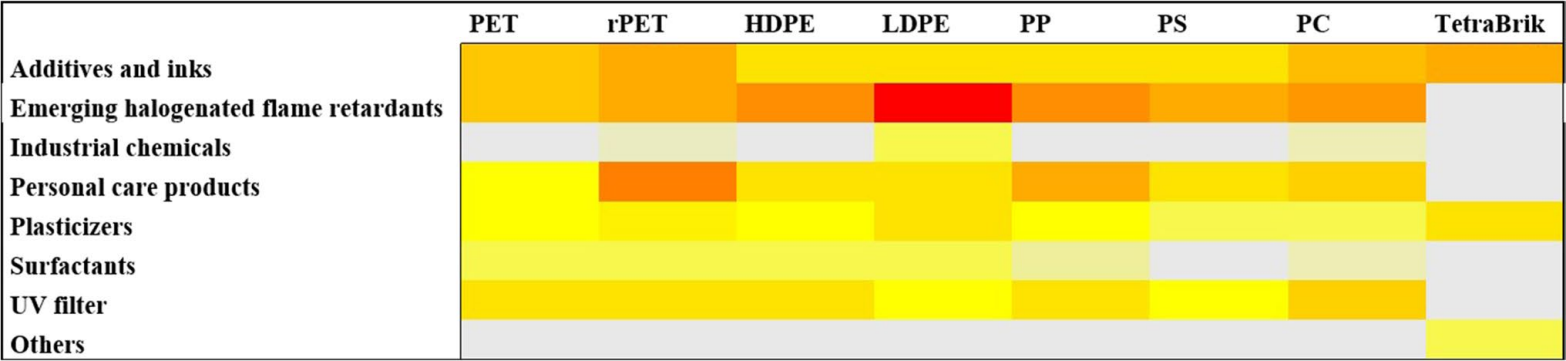
Heatmap of the identified chemical classes in the different material-based FCMs. The acronyms used for the FCMs are described in the “Experimental” section

None of the FCCs were identified exclusively in a specific type of FCM. However, the compounds identified in Tetra Brik were not common to any of the plastic-based FCMs. This aligns with the differing composition and manufacturing processes between plastic packaging and Tetra Brik. Among the eight chemical classes identified across all analyzed packaging types, the *emerging halogenated flame retardants* category contained the highest number of compounds and were present in all plastic-based packaging, as can be drawn from the heatmap shown in Fig. 6. Indeed, in negative ionization mode, only these FCCs, considered as NIAS of emerging concern, were detected. For instance, the butyl 2-ethoxyethyl hydrogen phosphate is a widely used compound in the field of FCMs, as evidenced by its high detection frequency (94% DF) despite being classified as a NIAS. Its primary applications focus on enhancing the flexibility, durability, and processibility of plastics such as polyethylene (PE) and polypropylene (PP). Additionally, it contributes to improving the barrier properties of FCMs, particularly against gases like oxygen and volatile compounds, thereby helping to preserve the quality of packaged foods. Our findings prove that emerging halogenated flame retardants are being increasingly used in FCMs, as replacement of traditional brominated flame retardants (BFRs). However, concerns about their potential health and environmental impacts persist, as many emerging halogenated flame retardants share similar properties to their predecessors. Different emerging halogenated flame retardants have been previously reported to migrate from FCMs, supporting the findings of this study [23].

*Additives and inks* followed as the second category with the highest number of identified compounds. Notably, compounds from this category were detected in all types of packaging analyzed, including both plastic-based FCMs and Tetra Brik, highlighting the importance of considering these compounds when assessing the risk of chemical exposure through FCMs. The third most prevalent category was *Personal care products*, which also contained compounds identified in all plastic-based packaging. Although this term typically refers to a diverse group of chemicals commonly found in cosmetics, skincare, and hygiene products, other substances such as fragrances and preservatives are also included in this category. These latter groups, in particular, are prone to being found in FCMs. For instance, methyl 2-(3-oxo-2-pentylcyclopentyl)acetate is the compound with the highest detection frequency within this category (89% DF), commonly used in the production of perfumes. However, it is also employed in the formulation of food-grade coatings, particularly for odor control purposes. It is worth noting that among the compounds identified in the three categories more frequently detected, only four of them are classified as IAS. This highlights the importance of assessing the presence of compounds other than IAS in FCMs. As shown in Figure S2, while other chemical categories are also present in multiple types of FCMs, these three categories collectively represent a detection frequency exceeding 50% of the total compounds identified for each FCM type.

The benzophenone-8, a widely used emerging *UV filter*, which is classified as IAS, has been detected with the highest detection frequency (94% DF) in positive ionization mode. UV filters are primarily employed to enhance the durability and stability of plastic packaging, coatings, and adhesives. *Plasticizers*, such as phthalates, are among the most frequently detected compounds in food packaging materials, as they are commonly used in paints, printing inks, lacquers, films, and adhesives [24]. Similarly, bisphenols are prevalent in recycled paper products, as they play a key role in the production of resins for printing ink formulations. Notably, the endocrine-disrupting chemical bisphenol A (BPA) was identified with a detection frequency of 50%. BPA, classified as an IAS, has been extensively documented in studies focusing on food contact materials, including those made from recycled sources.

When comparing PET-based FCMs with those made from recycled PET, compounds belonging to the same categories were identified, which is logical given that both are made from the same material. However, the number of compounds identified was higher in FCMs made from recycled material (Fig. 6), suggesting a concentration of contaminants through the cycle of the recycling and reuse processes. Thus, while 2 compounds classified as personal care have been identified in 1-PET FCMs, 5 different NIAS have been identified in the recycled one.

These results clearly illustrate that the developed SUPRAS-based method allows the detection of a wide variety of FCCs, covering a broad polarity range, and spanning multiple chemical classes with high detection frequencies. In contrast to conventional solvent-based methods, which often involve multiple repetitive extraction steps, the use of auxiliary energies, and large volumes of organic solvents [5, 11, 14], the SUPRAS approach achieves comprehensive extraction in a single, simple, and environmentally friendly step. This efficiency not only simplifies sample preparation but also enhances the likelihood of detecting a broader range of compounds, including emerging and previously unreported NIAS. The identification of 18 compounds not present in the FCCmigex database supports the method’s ability to overcome the limitations of traditional extraction strategies, enabling access to a wider and more representative chemical profile of FCMs.

## Conclusions

According to the results obtained, some significant insights can be drawn from this study. First, SUPRASs have been proven to be suitable for the development of sample treatment methods able to extract compounds covering a wide range of physicochemical properties (log P from − 5.2 to 9.8), which is considered crucial for addressing the wide chemical diversity present in FCMs. This capacity for developing all-in-one extractions, combined with the implementation of rigorous QA/QC protocols, the development of well-founded data analysis workflows, and the use of comprehensive in-house suspect lists for data reduction alongside the use of high-resolution techniques such as LC-HRMS is essential for ensuring reliable identification of FCCs when employing non-targeted analytical approaches. A total of 29 compounds were identified with a high degree of confidence, 18 of which were not included in the FCCmigex database. This underscores the value of adopting a holistic analytical strategy to uncover additional insights into the potential contribution of FCMs to human chemical exposure and highlights the critical need for continued investigation into the chemical safety of FCMs. Second, from an *operational* perspective, SUPRAS offer several benefits: they require no additional specialized equipment; their synthesis and applicability are straightforward, fast and cost-effective; and green amphiphilic molecules are widely available at low cost, offering high versatility. Third, *environmentally*, SUPRAS significantly reduce the ecological impact compared to conventional techniques. Their synthesis and extraction processes require minimal energy, eliminate or drastically reduce the use of organic solvents, and can be produced from biodegradable and/or renewable compounds. Additionally, the low volumes required generate minimal waste, further supporting their sustainability.

## Supplementary Information

Below is the link to the electronic supplementary material.

Supplementary file1 (DOCX 711 KB)

## Data Availability

No datasets were generated or analysed during the current study.
