# Supplementary material for: Supramolecular solvent-based all-in-one extractions for comprehensive suspect screening of chemicals in food contact materials
Source: Mikrochim Acta. 2025 Jun 3;192(7):398. doi: 10.1007/s00604-025-07216-8 (PMC12133993; doi:10.1007/s00604-025-07216-8)
Supplement: Supplementary file 1 — Supplementary file1 (DOCX 711 KB) [file 604_2025_7216_MOESM1_ESM.docx]

**Supramolecular solvent-based all-in-one extractions for comprehensive suspect screening of chemicals in food contact materials**

Laura García-Cansino^a,b^, Noelia Caballero-Casero*^b^, María Ángeles García^a,c^, María Luisa Marina^a,c^, Soledad Rubio^b^

^a^Universidad de Alcalá, Departamento de Química Analítica, Química Física e Ingeniería Química, Ctra. Madrid-Barcelona Km. 33.600, 28871 Alcalá de Henares (Madrid), Spain.

^b^Department of Analytical Chemistry, Institute of Chemistry for Energy and the Environment, Anexo Marie Curie, Campus de Rabanales, Universidad de Córdoba (Córdoba), 14071, Spain.

^c^Universidad de Alcalá, Instituto de Investigación Química Andrés M. del Río, Ctra. Madrid-Barcelona Km. 33.600, 28871Alcalá de Henares (Madrid), Spain.

***Corresponding author**: email: a42caasn@uco.es, Phone +34 957 212441

García-Cansino ORCID: 0000-0003-2531-1967

Caballero-Casero ORCID: 0000-0002-0662-1815

García ORCID: 0000-0001-6292-8232

Marina ORCID: 0000-0002-5583-1624

Rubio ORCID: 0000-0002-9128-4787

**TABLE OF CONTENTS**

FIGURES

Figure S1. Schematic diagram of the synthesis of SUPRASs ………………….…...…..3

Figure S2. Bar chart illustrating the contribution of each chemical group to the total number of compounds identified in each type of FCM studied…………………………4

TABLES

Table S1. Information on the isotopically-labelled IS used………………………...……5

Table S2. Experimental conditions for the synthesis of the SUPRASs investigated….....6

**Figure S1.** Schematic diagram of the synthesis of SUPRASs through a bottom-up approach based on coacervation.


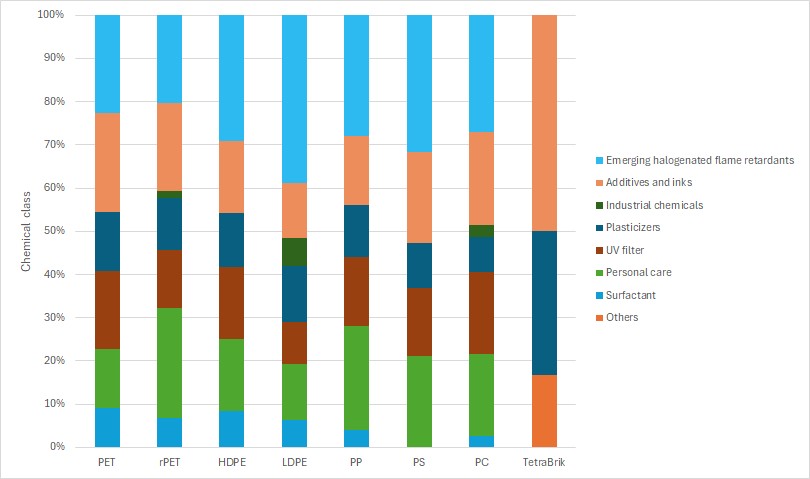


**Figure S2**. Bar chart illustrating the contribution of each chemical group to the total number of compounds identified in each type of FCM studied.

**Table S1.** Chemical classes of the labeled-IS used in the study with their IUPAC chemical names and abbreviations, chemical structures, formula, monoisotopic mass, partition coefficient (log P) and ionization mode in electrospray ion source.

**Table S2.** Relative proportions of reagents and solvents used in the synthesis of the four SUPRASs investigated for extraction of FCCs.

| **Type of SUPRAS** | **Amphiphile (%)** | **Solvent (%)** | **Coacervation-inducing agent (%)** |
| --- | --- | --- | --- |
| SUPRAS 1 | 1,2-decanediol  (15% w/w) | THF  (15%, w/w) | Aqueous 1 M NaCl  (70%, w/w) |
| SUPRAS 2 | 1,2-decanediol  (15% w/w) | THF  (15%, w/w) | Water  (70%, w/w) |
| SUPRAS 3 | 1-decanol  (10% v/v) | Ethanol  (30%, v/v) | Water  (60%, v/v) |
| SUPRAS 4 | 1-octanol  (10% v/v) | THF  (30%, v/v) | Water  (60%, v/v) |
